# Association Between Periodontal Status and Undiagnosed Diabetes in Adults Without Diagnosed Chronic Diseases: The Korea National Health and Nutrition Examination Survey 2013–2015

**DOI:** 10.3390/jcm15197747

**Published:** 2026-10-08

**Authors:** Young Sang Lyu, So Yeong Kim, Sang Hee Jeong, Youngmin Yoon, Sang Yong Kim, Jin Hwa Kim

**Affiliations:** 1 Department of Endocrinology and Metabolism, Chosun University Hospital, Chosun University College of Medicine, Gwangju 61453, Republic of Korea; 2 Department of Preventive Medicine, Chosun University College of Medicine, Gwangju 61453, Republic of Korea; 3 Department of Internal Medicine, Chosun University Hospital, Chosun University College of Medicine, Gwangju 61453, Republic of Korea; 4 Division of Nephrology, Department of Internal Medicine, Chosun University Hospital, Chosun University College of Medicine, Gwangju 61453, Republic of Korea

**Keywords:** periodontitis, Community Periodontal Index, undiagnosed diabetes, prediabetes, cross-sectional study, KNHANES

## Abstract

**Background/Objectives:** Periodontitis and diabetes are closely linked; however, whether periodontal findings are associated with previously unrecognized dysglycemia in adults without known chronic diseases remains unclear. We examined the association between periodontal status and undiagnosed prediabetes and diabetes in this population. **Methods:** We analyzed data from 7635 adults (3218 men and 4417 women) aged ≥19 years without self-reported physician-diagnosed chronic diseases who participated in the Korea National Health and Nutrition Examination Survey 2013–2015. Glycemic status was classified as normal, undiagnosed prediabetes, or undiagnosed diabetes based on a single measurement of fasting plasma glucose and glycated hemoglobin. Periodontal status was determined with the Community Periodontal Index (CPI) and grouped as CPI 0–2, CPI 3, or CPI 4. Survey-weighted multivariable logistic regression analyses were performed in men and women separately, and effect modification by sex was formally tested. **Results:** Undiagnosed prediabetes and diabetes were present in 42.0% and 5.0% of men and in 31.7% and 2.9% of women (weighted estimates). After multivariable adjustment, CPI 3 (odds ratio [OR] 3.594, 95% confidence interval [CI] 1.553–8.316) and CPI 4 (OR 5.837, 95% CI 2.341–14.555) were associated with higher odds of undiagnosed diabetes in men, whereas no significant association was observed in women (*p* for sex interaction = 0.004). Periodontal status was not associated with undiagnosed prediabetes in either sex. **Conclusions:** In this cross-sectional analysis, CPI-defined periodontitis was associated with higher odds of undiagnosed diabetes in men. These findings do not establish causality or the diagnostic performance of periodontal examination as a screening test; rather, periodontal findings may help identify individuals in whom standard diabetes risk assessment or medical referral could be considered.

## 1. Introduction

Diabetes is becoming increasingly common worldwide, yet many people who have it are not aware of their condition. The IDF Diabetes Atlas estimated that 537 million adults aged 20–79 years (10.5%) were living with diabetes in 2021 and that almost half of these cases were undiagnosed [1]. When diagnosis is delayed, hyperglycemia persists untreated, which can hasten the onset of diabetic complications and raise the likelihood of atherosclerotic cardiovascular disease [2,3].

Periodontitis is a common chronic inflammatory condition in which the tissues that support the teeth are progressively destroyed. Severe forms that threaten tooth retention are found in roughly 10–15% of adults in most surveyed populations, and up to 90% of adults are affected when milder forms are also counted [4,5]. The disease arises from a dysbiotic oral microbiota that provokes a dysregulated host immune response [6]; although this response begins at the tooth surface, it also adds to the overall inflammatory load of the body [7,8]. This systemic inflammatory burden has been linked to several chronic conditions, among them cardiovascular disease, chronic kidney disease, and type 2 diabetes mellitus (T2DM) [7,8,9,10]. Notably, consensus evidence supports an association between periodontitis and atherosclerotic cardiovascular disease, and periodontitis has also been linked to chronic kidney disease [11,12]. The relationship between periodontitis and diabetes should therefore be viewed within the broader framework of periodontal–systemic interactions.

Of these conditions, T2DM shows one of the most consistent links with periodontitis. Cross-sectional data indicate that diabetes is more common among people with periodontitis [13,14], and a randomized trial showed that intensive treatment of moderate-to-severe periodontitis lowered glycated hemoglobin (HbA1c) in people with T2DM [15]. However, most previous studies were relatively small or were conducted in selected clinical populations, and large population-based evidence on whether periodontal status is associated with previously unrecognized dysglycemia remains limited. Clarifying this association in adults who are unaware of having any chronic disease is clinically relevant, because dental visits may offer an opportunity to identify individuals who could benefit from standard diabetes assessment. Therefore, we examined the association between periodontal status, assessed using the Community Periodontal Index (CPI), and undiagnosed prediabetes and diabetes in a nationally representative sample of Korean adults without diagnosed chronic diseases.

## 2. Materials and Methods

### 2.1. Study Population

We used data from the sixth Korea National Health and Nutrition Examination Survey (KNHANES VI, 2013–2015), a nationwide cross-sectional survey conducted by the Korea Disease Control and Prevention Agency (formerly the Korea Centers for Disease Control and Prevention) under the Ministry of Health and Welfare. Survey participants are drawn from community-dwelling Korean residents through multistage sampling stratified by region, sex, and age, and each participant undergoes an interview, a physical and laboratory examination, and a dietary survey conducted by trained staff. All KNHANES participants provided written informed consent, and the survey was conducted in accordance with the Declaration of Helsinki. The protocol for this secondary analysis received approval from the Chosun University Hospital Institutional Review Board (No. 2022-04-043).

**Figure 1:**
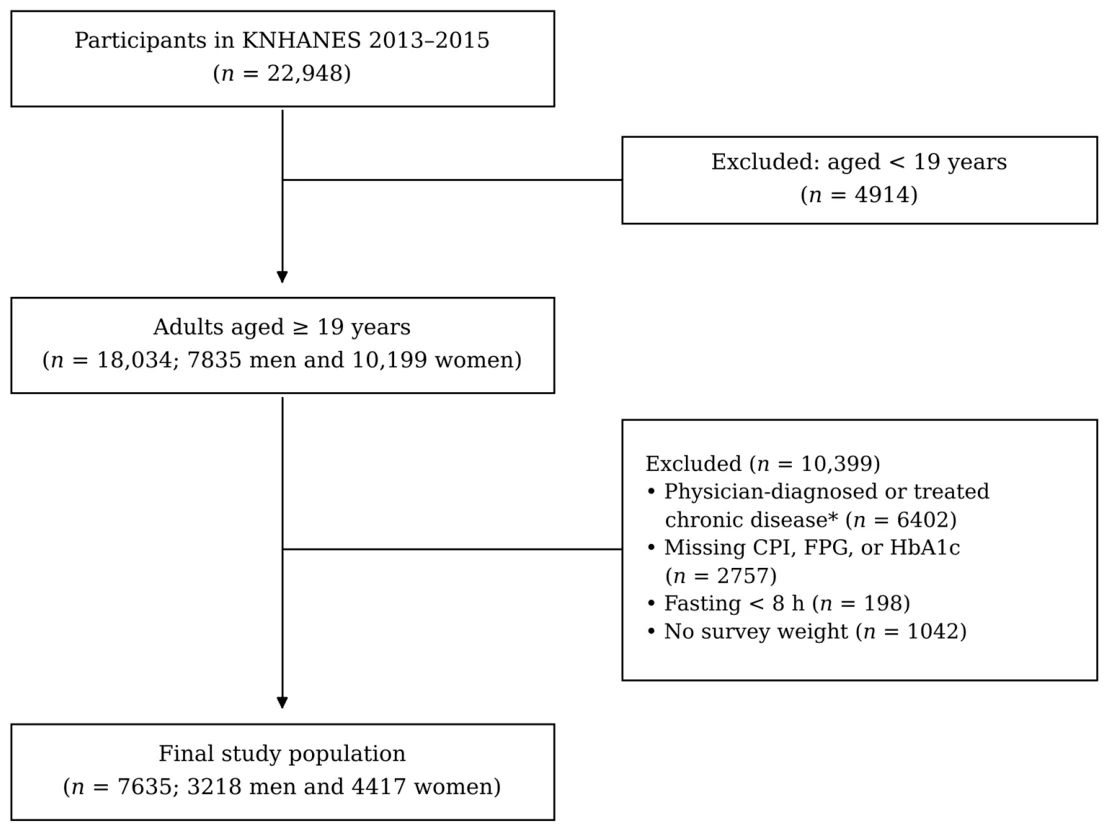
Flow diagram of the selection of study participants. * Hypertension, diabetes, dyslipidemia, coronary artery disease, cerebral infarction, chronic kidney or liver failure, or malignancy. FPG, fasting plasma glucose; HbA1c, glycated hemoglobin; KNHANES, Korea National Health and Nutrition Examination Survey.

Of the 22,948 individuals who participated in KNHANES 2013–2015, 18,034 adults aged ≥19 years (7835 men and 10,199 women) were considered for inclusion. We excluded participants who reported a physician diagnosis of, or current treatment for, hypertension, diabetes, dyslipidemia, coronary artery disease, cerebral infarction, chronic kidney or liver failure, or malignancy (n = 6402). These exclusions were specified a priori for two reasons: first, to restrict the analysis to individuals who were unaware of having major chronic diseases, so that any dysglycemia detected in the survey could be regarded as previously unrecognized; and second, to minimize confounding by chronic diseases and their treatments (e.g., antihypertensive, lipid-lowering, and glucose-lowering agents) that may affect both periodontal health and glucose metabolism. We further excluded participants with missing data on the periodontal examination, fasting plasma glucose (FPG), or HbA1c (n = 2757), those with a fasting time shorter than 8 h (n = 198), and those without a survey weight, who could not be included in the design-based analyses (n = 1042). Consequently, 7635 adults (3218 men and 4417 women) were included in the final analysis (Figure 1). Because the study population was defined by the absence of self-reported diagnosed chronic diseases, participants may still have had other diagnosed or undiagnosed conditions; we therefore refer to them as adults without diagnosed chronic diseases rather than as healthy individuals.

### 2.2. Measurements, Covariates, and Glycemic Status

Standardized KNHANES procedures were used for all measurements. Body weight (balance scale) and height (portable stadiometer) were measured, and BMI was derived as weight (kg)/height (m)^2^. After participants had fasted overnight for at least 8 h, venous blood was drawn; glucose was quantified on a Hitachi 7600 automated analyzer (Hitachi, Tokyo, Japan), and HbA1c was quantified by high-performance liquid chromatography on a Tosoh G8 system (Tosoh, Tokyo, Japan).

Sociodemographic and lifestyle information was collected using standardized questionnaires. Place of residence (urban or rural) was assigned from the administrative district. Educational attainment was dichotomized as high school graduation or higher vs. lower, and household income was categorized into quartiles. Smoking status was classified as never (never smoked), past (smoked previously but not currently), or current (currently smoking daily or occasionally). Alcohol drinking was defined as drinking alcohol at least once a month during the past year. Participants were considered to exercise regularly if they reported at least 150 min/week of moderate-intensity or 75 min/week of vigorous-intensity aerobic activity, or an equivalent combination. Tooth-brushing frequency was dichotomized as ≥3 or <3 times per day.

Glycemic status was classified using FPG and HbA1c values measured on the same occasion, according to the American Diabetes Association (ADA) criteria [16]. Participants with FPG ≥ 126 mg/dL or HbA1c ≥ 6.5% were classified as having undiagnosed diabetes; those with FPG 100–125 mg/dL or HbA1c 5.7–6.4% who did not meet the diabetes criteria, as having undiagnosed prediabetes; and those with FPG < 100 mg/dL and HbA1c ≤ 5.6%, as having normal glycemia. Because HbA1c values in KNHANES are reported to one decimal place, the prediabetes range of HbA1c > 5.6% and < 6.5% is equivalent to 5.7–6.4%. The three categories were mutually exclusive and assigned hierarchically: participants who met either criterion for diabetes were classified as having diabetes regardless of the other measure, and those with discordant values below the diabetes thresholds were classified as having prediabetes if either FPG or HbA1c was in the prediabetic range. Because classification was based on a single measurement without confirmatory repeat testing, it represents an epidemiological classification rather than a definitive clinical diagnosis.

### 2.3. Assessment of Periodontal Status

Oral examinations were carried out by dentists who had undergone standardized calibration. Following the World Health Organization CPI method, examiners used a CPI probe with a 0.5 mm ball end, applied light and uniform force, and examined the index teeth of each of the six sextants (substituting the other teeth of that sextant if the index tooth was missing). Each sextant received the worst code found: code 0 (healthy), code 1 (bleeding on probing), code 2 (calculus), code 3 (pocket depth 3.5–5.5 mm), or code 4 (pocket depth > 5.5 mm), and the maximum code among all sextants defined the periodontal status of the participant. For the analysis, participants were grouped into CPI 0–2 (no periodontal pocketing; reference), CPI 3 (shallow periodontal pocketing), and CPI 4 (deep periodontal pocketing), and CPI ≥ 3 was regarded as CPI-defined periodontitis. Because the CPI is an epidemiological index based on pocket depth, it does not capture clinical attachment loss, radiographic bone loss, or disease extent and does not correspond to the staging and grading framework of the 2018 classification of periodontitis [17]. Accordingly, CPI categories in this study were interpreted as indicators of periodontal pocketing rather than as clinical diagnoses of periodontitis stage or severity.

### 2.4. Statistical Analysis

To reflect the multistage design of KNHANES, sampling weights, strata, and primary sampling units were specified in the SPSS complex samples module, as recommended by the analytic guidelines of the Korea Disease Control and Prevention Agency; these design variables were retained in every model, including the sex-stratified analyses. Continuous variables are presented as weighted means ± standard errors (SEs), and categorical variables as unweighted numbers with weighted percentages. Group comparisons were based on design-based general linear models for continuous data and design-adjusted chi-square tests for categorical data.

Survey-weighted multinomial logistic regression analyses, with normal glycemia as the reference outcome category, were used to estimate odds ratios (ORs) and 95% confidence intervals (CIs) for undiagnosed prediabetes and undiagnosed diabetes according to periodontal status, with CPI 0–2 as the reference category. Three models were constructed: Model 1 was unadjusted; Model 2 was adjusted for age; and Model 3 was further adjusted for BMI, smoking status, alcohol drinking, household income, education, residential area, regular exercise, and tooth-brushing frequency. Age and BMI were entered as continuous variables, and the remaining covariates as categorical variables. Covariates were chosen in advance because they are known or plausible determinants of both periodontal health and glycemia. Linear trends across CPI categories were tested by entering periodontal status as an ordinal variable in the models. Because the prevalence of periodontitis and undiagnosed dysglycemia differs by sex, all analyses were stratified by sex. In addition, to formally assess whether the association differed between men and women, a sex × periodontal status interaction term was added to Model 3 in the total study population [18]. Sensitivity analyses were performed using alternative definitions of glycemic status: (1) FPG criteria only; (2) HbA1c criteria only; and (3) the World Health Organization threshold for impaired fasting glucose (FPG 110–125 mg/dL) for prediabetes. Participants with missing covariate data were excluded from the regression models (complete-case analysis). Because regular aerobic exercise was assessed only in 2014 and 2015, the regression models included participants from these two survey years with complete covariate data (men, n = 1817; women, n = 2536), and the same analytic sample was used for Models 1–3. Analyses were run in SPSS 25.0 (IBM Corp., Armonk, NY, USA), with statistical significance set at a two-sided *p* < 0.05. This study is reported in accordance with the Strengthening the Reporting of Observational Studies in Epidemiology (STROBE) guidelines [19].

## 3. Results

### 3.1. Characteristics by Periodontal Status

Table 1 presents the characteristics of men according to periodontal status. The weighted proportions of men with CPI 3 and CPI 4 were 17.0% and 16.6%, respectively. Compared with men with CPI 0–2, those with CPI 4 were older, had a slightly lower BMI, were more likely to be current or past smokers, and had lower household income and educational attainment; they were also less likely to brush their teeth at least three times per day, to exercise regularly, and to live in urban areas. Alcohol drinking did not differ significantly across the groups. FPG and HbA1c levels were higher in men with CPI 3 or CPI 4 than in those with CPI 0–2, and the weighted prevalence of undiagnosed diabetes was 2.4%, 8.6%, and 11.9% in the CPI 0–2, CPI 3, and CPI 4 groups, respectively.

The corresponding characteristics of women are shown in Table 2. The weighted proportions of women with CPI 3 and CPI 4 were 12.4% and 11.4%, respectively. Women with CPI 4 were older and had a higher BMI and lower household income and educational attainment; alcohol drinking, tooth brushing at least three times daily, regular exercise, and urban residence were all less common in this group (all *p* < 0.05). As in men, FPG and HbA1c levels were higher in the CPI 3 and CPI 4 groups, and the weighted prevalence of undiagnosed diabetes was 2.1%, 3.6%, and 7.8% in the CPI 0–2, CPI 3, and CPI 4 groups, respectively.

### 3.2. Characteristics by Glycemic Status

Table 3 compares the characteristics of men according to glycemic status. Relative to men with normal glycemia, men with undiagnosed diabetes were older and had higher BMI, were more likely to smoke, had lower educational attainment, and were less likely to brush their teeth at least three times per day or to exercise regularly. The weighted proportion of men with CPI 3 or CPI 4 was 67.6% among those with undiagnosed diabetes, compared with 24.0% among those with normal glycemia.

Table 4 presents the corresponding comparison in women. Women with undiagnosed diabetes were older and had higher BMI, lower household income, and lower educational attainment, and they less often reported alcohol drinking, tooth brushing at least three times daily, or regular exercise. Smoking status did not differ significantly across the groups. The weighted proportion of women with CPI 3 or CPI 4 was 45.7% among those with undiagnosed diabetes, compared with 17.2% among those with normal glycemia.

### 3.3. Association Between Periodontal Status and Undiagnosed Prediabetes and Diabetes

Table 5 presents the numbers of cases and weighted prevalence among participants included in the regression models, together with the fully adjusted ORs for undiagnosed prediabetes and diabetes according to periodontal status; the results of the unadjusted and age-adjusted models are shown in Supplementary Table S1. In men, CPI 3 and CPI 4 were associated with higher odds of undiagnosed diabetes compared with CPI 0–2 after full adjustment (CPI 3: OR 3.594, 95% CI 1.553–8.316; CPI 4: OR 5.837, 95% CI 2.341–14.555; *p* for trend < 0.001). The ORs were attenuated after adjustment for age (Model 1: CPI 3, OR 7.378 and CPI 4, OR 15.579; Model 2: 4.713 and 6.169, respectively) but remained statistically significant in the fully adjusted model (Supplementary Table S1). Because the numbers of cases entering the regression models were small (18, 24, and 51 men with undiagnosed diabetes in the CPI 0–2, CPI 3, and CPI 4 groups, respectively), the CIs were wide, and the magnitude of these estimates should be interpreted with caution. Periodontal status was not significantly associated with undiagnosed prediabetes in men.

In women, neither undiagnosed prediabetes nor undiagnosed diabetes showed a significant association with periodontal status in the fully adjusted model (*p* for trend = 0.936 for undiagnosed diabetes). In the total study population, the interaction between sex and periodontal status was statistically significant for undiagnosed diabetes (*p* for interaction = 0.004) but not for undiagnosed prediabetes (*p* for interaction = 0.916). In sensitivity analyses using alternative definitions of glycemic status, the results for undiagnosed diabetes were consistent with the main findings, with significant associations in men and no significant associations in women (Supplementary Table S2).

## 4. Discussion

In this nationally representative sample of Korean adults without diagnosed chronic diseases, the weighted prevalence of undiagnosed prediabetes and diabetes was higher among individuals with CPI-defined periodontitis, and individuals with undiagnosed dysglycemia more frequently had periodontal pocketing. After adjustment for potential confounders, CPI 3 and CPI 4 were associated with higher odds of undiagnosed diabetes in men, whereas no significant association was observed in women. Periodontal status was not associated with undiagnosed prediabetes in either sex. Given the cross-sectional design, these findings indicate associations rather than causal effects.

In 2022, the ADA lowered the recommended age for initiating diabetes screening in asymptomatic adults from 45 to 35 years [16]. The change was prompted by the rising number of younger adults with diabetes and by simulation studies suggesting that finding and treating diabetes earlier could lower cardiovascular events and deaths [1,20]. Previous studies conducted in dental settings have shown that a considerable proportion of dental patients have unrecognized dysglycemia and that simple dental and clinical parameters can help identify such individuals [21,22,23]. These studies, however, were modest in size, and most did not consider the comorbid conditions of participants. Our findings extend this evidence to a nationally representative sample restricted to adults without diagnosed chronic diseases. Nevertheless, we did not evaluate the screening performance of periodontal examination, such as its sensitivity, specificity, predictive values, or discriminative ability; therefore, our results should not be interpreted as evidence that periodontal examination is a valid screening test for diabetes.

Men in our study had a higher prevalence of both periodontitis and undiagnosed diabetes than women, in line with earlier reports [24,25]. The higher rates of smoking and alcohol drinking among men, both of which are established risk factors for periodontitis, may partly explain this difference. Notably, these findings suggest that unrecognized dysglycemia is not uncommon even among men who have not been diagnosed with any chronic disease.

The association between periodontal status and undiagnosed diabetes was statistically significant in men but not in women. However, a significant association in one sex and a non-significant association in the other does not by itself indicate a true difference between the sexes. The formal interaction test supported effect modification by sex (*p* for interaction = 0.004), although this finding requires replication given the relatively small number of cases. Possible explanations for a sex difference, such as sex differences in fat distribution and insulin sensitivity [26] and the reported effects of estrogen on periodontal tissues [27], remain speculative because sex hormones and menopausal status were not assessed in this study. This issue should be examined in adequately powered studies with hormonal data.

In men, CPI 3 and CPI 4 were associated with approximately 3.6-fold and 5.8-fold higher odds of undiagnosed diabetes, respectively, although the wide CIs reflect the limited number of cases. This finding is consistent with the well-described bidirectional relationship between periodontitis and diabetes. Diabetes is a well-recognized risk factor for periodontitis and is estimated to roughly triple its risk [28,29,30,31]. Conversely, periodontitis has been associated with poorer glycemic control [10,32,33], and periodontal treatment has been shown to reduce HbA1c levels by approximately 0.3–0.5% in people with diabetes [34].

Periodontal status was associated with undiagnosed diabetes but not with undiagnosed prediabetes. One possible explanation is that measurable periodontal tissue destruction may become evident only after more prolonged or pronounced hyperglycemia; alternatively, the heterogeneity of the prediabetes category, defined using single measurements, may have diluted any association. By contrast, Tan et al. observed a dose–response relationship between periodontitis and prediabetes in a large population-based sample [35], which suggests that this relationship may vary across populations and diagnostic criteria.

Importantly, our cross-sectional data cannot reveal which condition came first, and reverse causation is at least as plausible as the alternative. Prolonged undiagnosed hyperglycemia may have contributed to periodontal tissue destruction through impaired host defense and enhanced inflammation, whereas periodontal inflammation may, in turn, aggravate insulin resistance [36]. Longitudinal studies are required to clarify the temporal sequence of these conditions.

Several mechanisms may underlie this association, although none of them were measured in the present study. Alterations in the oral microbiome have been reported in individuals with diabetes and periodontitis [37,38], and chronic hyperglycemia and periodontal inflammation share proinflammatory pathways involving cytokines such as tumor necrosis factor-α and interleukin-6 [5,39]. In addition, the two conditions share socioeconomic and behavioral risk factors, including low income, low educational attainment, smoking, and obesity. Although we adjusted for these factors, residual confounding cannot be excluded.

Key strengths of this work are its nationwide, population-representative sample, the standardized dental and laboratory examinations, and the design-based analytic approach. However, several limitations should be considered. First, the cross-sectional design precludes inferences about causality or temporal direction. Second, periodontal status was assessed using the CPI, which is based on pocket depth at index teeth and does not incorporate clinical attachment loss, radiographic bone loss, or disease extent. Therefore, CPI categories do not correspond to the staging and grading framework of the 2018 classification [17], and misclassification of periodontal status is likely [40]. Because such misclassification is probably non-differential with respect to glycemic status, it would tend to bias the associations toward the null; however, differential misclassification cannot be completely ruled out. Third, glycemic status was based on a single measurement of FPG and HbA1c without confirmatory testing; thus, some participants may have been misclassified, and our categories do not constitute clinical diagnoses. Fourth, we did not measure inflammatory markers, sex hormones, or the oral microbiome, and residual confounding by unmeasured factors is possible. Fifth, lifestyle and oral hygiene behaviors were self-reported and may be affected by recall errors or socially desirable answering. Sixth, the exclusion of participants with diagnosed chronic diseases, although intended to identify individuals with unrecognized dysglycemia and to reduce confounding, limits the generalizability of our findings to the general adult population and may have introduced selection bias. In addition, the number of participants with undiagnosed diabetes was small, particularly among women, resulting in wide CIs. Finally, the data were collected in 2013–2015 from a Korean population; therefore, the prevalence estimates may not reflect current conditions, and the findings may not be generalizable to other ethnic groups or healthcare settings.

From a clinical perspective, periodontal examination is routinely performed during dental visits, which are frequently attended by individuals who do not undergo regular medical checkups. Our findings suggest that CPI-defined periodontal findings, particularly in men, may help identify individuals in whom standard diabetes risk assessment or referral for FPG or HbA1c testing could be considered. However, whether incorporating periodontal status into diabetes risk assessment improves the detection of diabetes, and whether such an approach is clinically effective or cost-effective, was not examined in this study and should be evaluated in prospective studies that formally assess screening performance.

## 5. Conclusions

In this cross-sectional analysis of Korean adults without diagnosed chronic diseases, CPI-defined periodontitis was associated with higher odds of undiagnosed diabetes in men. These findings do not establish causality or the diagnostic performance of periodontal examination as a screening test for diabetes. Rather, periodontal findings may help identify individuals in whom standard diabetes risk assessment or medical referral could be considered. Prospective studies are needed to clarify the temporal relationship between periodontitis and diabetes and to evaluate the clinical utility of this approach.

## Figures and Tables

**Table 1:** Participant characteristics by CPI category: men.

	CPI 0–2	CPI 3	CPI 4	*p*-Value
Number, n (weighted %)	1926 (66.4)	572 (17.0)	720 (16.6)	
Age (years)	35.63 ± 0.30	45.58 ± 0.52	55.52 ± 0.55	0.001
BMI (kg/m^2^)	24.19 ± 0.09	24.51 ± 0.16	23.99 ± 0.13	0.001
Tooth brushing ≥ 3 times/day (%)	948 (49.6)	252 (45.2)	271 (38.1)	0.001
Family income percentile (%)				0.001
<25	140 (7.3)	60 (8.5)	174 (19.5)	
25–50	455 (23.6)	143 (25.1)	197 (26.9)	
50–75	630 (32.7)	183 (34.8)	184 (27.6)	
≥75	701 (36.4)	184 (31.6)	160 (26.0)	
Education ≥ high school (%)	1640 (87.5)	402 (75.4)	346 (54.9)	0.001
Urban residence (%)	1641 (86.3)	460 (81.5)	500 (72.6)	0.001
Smoking				0.001
Never	567 (33.2)	84 (15.9)	87 (10.8)	
Past	573 (28.1)	191 (32.7)	286 (38.7)	
Current	690 (38.8)	261 (51.4)	288 (50.5)	
Alcohol drinking (Yes, %)	1392 (76.4)	397 (74.0)	458 (72.8)	0.255
Regular exercise (Yes, %) ^a^	689 (65.1)	168 (53.4)	209 (51.3)	0.001
AST (IU/L)	22.72 ± 0.27	24.61 ± 0.60	24.86 ± 0.49	0.001
ALT (IU/L)	26.68 ± 0.64	28.75 ± 1.07	24.08 ± 0.62	0.001
FPG (mg/dL)	94.08 ± 0.31	101.84 ± 1.15	102.53 ± 0.95	0.001
HbA1c (%)	5.50 ± 0.01	5.74 ± 0.04	5.85 ± 0.03	0.001
Glycemic status				0.001
Normal	1101 (60.6)	223 (39.0)	228 (33.3)	
Undiagnosed prediabetes ^b^	771 (37.0)	300 (52.4)	404 (54.7)	
Undiagnosed diabetes ^c^	54 (2.4)	49 (8.6)	88 (11.9)	

Values are weighted mean ± standard error (continuous variables) or unweighted n (weighted %) (categorical variables). *p*-Values were obtained from design-based general linear models (continuous variables) or design-adjusted chi-square tests (categorical variables). ALT, alanine aminotransferase; AST, aspartate aminotransferase; BMI, body mass index; CPI, Community Periodontal Index; FPG, fasting plasma glucose; HbA1c, glycated hemoglobin. ^a^ At least 150 min/week of moderate-intensity or 75 min/week of vigorous-intensity aerobic activity, or an equivalent combination. ^b^ FPG 100–125 mg/dL or HbA1c 5.7–6.4%, without meeting the criteria for diabetes. ^c^ FPG ≥ 126 mg/dL or HbA1c ≥ 6.5%.

**Table 2:** Participant characteristics by CPI category: women.

	CPI 0–2	CPI 3	CPI 4	*p*-Value
Number, n (weighted %)	3206 (76.3)	567 (12.4)	644 (11.4)	
Age (years)	37.44 ± 0.26	46.88 ± 0.65	57.83 ± 0.70	0.001
BMI (kg/m^2^)	22.44 ± 0.07	23.44 ± 0.16	23.81 ± 0.17	0.001
Tooth brushing ≥ 3 times/day (%)	1980 (62.8)	303 (55.5)	261 (43.5)	0.001
Family income percentile (%)				0.001
<25	257 (7.6)	71 (10.9)	212 (28.3)	
25–50	760 (23.6)	162 (29.4)	159 (24.3)	
50–75	1025 (32.2)	166 (29.9)	143 (25.3)	
≥75	1151 (36.6)	163 (29.8)	120 (22.1)	
Education ≥ high school (%)	2628 (84.7)	362 (67.0)	218 (40.4)	0.001
Urban residence (%)	2772 (88.0)	443 (79.9)	446 (73.3)	0.001
Smoking				0.085
Never	2758 (87.8)	483 (89.4)	524 (88.5)	
Past	191 (6.5)	17 (3.4)	28 (5.3)	
Current	152 (5.8)	40 (7.3)	32 (6.2)	
Alcohol drinking (Yes, %)	1516 (51.2)	215 (40.1)	199 (36.4)	0.001
Regular exercise (Yes, %) ^a^	1012 (56.6)	166 (51.8)	144 (40.1)	0.001
AST (IU/L)	18.53 ± 0.15	20.36 ± 0.47	21.43 ± 0.36	0.001
ALT (IU/L)	15.43 ± 0.24	17.53 ± 0.62	18.23 ± 0.48	0.001
FPG (mg/dL)	91.78 ± 0.30	95.71 ± 0.78	97.12 ± 0.66	0.001
HbA1c (%)	5.47 ± 0.01	5.61 ± 0.03	5.73 ± 0.03	0.001
Glycemic status				0.001
Normal	2152 (70.9)	281 (52.5)	261 (42.1)	
Undiagnosed prediabetes ^b^	974 (27.0)	257 (43.9)	331 (50.1)	
Undiagnosed diabetes ^c^	80 (2.1)	29 (3.6)	52 (7.8)	

Values are weighted mean ± standard error (continuous variables) or unweighted n (weighted %) (categorical variables). *p*-Values were obtained from design-based general linear models (continuous variables) or design-adjusted chi-square tests (categorical variables). ALT, alanine aminotransferase; AST, aspartate aminotransferase; BMI, body mass index; CPI, Community Periodontal Index; FPG, fasting plasma glucose; HbA1c, glycated hemoglobin. ^a^ At least 150 min/week of moderate-intensity or 75 min/week of vigorous-intensity aerobic activity, or an equivalent combination. ^b^ FPG 100–125 mg/dL or HbA1c 5.7–6.4%, without meeting the criteria for diabetes. ^c^ FPG ≥ 126 mg/dL or HbA1c ≥ 6.5%.

**Table 3:** Participant characteristics by glycemic status: men.

	Normal	Undiagnosed Prediabetes ^b^	Undiagnosed Diabetes ^c^	*p*-Value
Number, n (weighted %)	1552 (53.0)	1475 (42.0)	191 (5.0)	
Age (years)	36.07 ± 0.37	45.10 ± 0.38	51.08 ± 0.89	0.001
BMI (kg/m^2^)	23.63 ± 0.09	24.79 ± 0.10	25.63 ± 0.27	0.001
Tooth brushing ≥ 3 times/day (%)	751 (49.8)	649 (44.5)	71 (39.0)	0.005
Family income percentile (%)				0.940
<25	170 (9.4)	177 (9.3)	33 (12.0)	
25–50	376 (24.1)	364 (24.6)	55 (25.6)	
50–75	461 (32.6)	479 (32.3)	50 (28.7)	
≥75	520 (34.2)	467 (33.5)	53 (33.7)	
Education ≥ high school (%)	1270 (86.2)	1030 (74.2)	124 (64.5)	0.001
Urban residence (%)	1291 (85.8)	1163 (80.4)	147 (79.2)	0.001
Smoking				0.001
Never	442 (32.2)	269 (20.8)	27 (14.5)	
Past	472 (28.2)	514 (33.1)	64 (35.5)	
Current	576 (39.7)	589 (46.1)	74 (50.0)	
Alcohol drinking (Yes, %)	1147 (73.9)	1041 (76.9)	126 (79.1)	0.166
Regular exercise ^a^ (Yes, %)	617 (65.6)	398 (54.0)	51 (54.7)	0.001
AST (IU/L)	22.33 ± 0.32	24.32 ± 0.31	27.03 ± 1.42	0.001
ALT (IU/L)	24.55 ± 0.72	28.49 ± 0.67	32.45 ± 1.85	0.001
FPG (mg/dL)	89.46 ± 0.18	100.10 ± 0.28	146.80 ± 3.25	0.001
HbA1c (%)	5.31 ± 0.01	5.76 ± 0.01	7.29 ± 0.10	0.001
Periodontal status				0.001
CPI 0–2	1101 (76.0)	771 (58.5)	54 (32.2)	
CPI 3	223 (13.6)	300 (20.0)	49 (28.1)	
CPI 4	228 (10.4)	404 (21.6)	88 (39.5)	

Values are weighted mean ± standard error (continuous variables) or unweighted n (weighted %) (categorical variables). *p*-Values were obtained from design-based general linear models (continuous variables) or design-adjusted chi-square tests (categorical variables). ALT, alanine aminotransferase; AST, aspartate aminotransferase; BMI, body mass index; CPI, Community Periodontal Index; FPG, fasting plasma glucose; HbA1c, glycated hemoglobin. ^a^ At least 150 min/week of moderate-intensity or 75 min/week of vigorous-intensity aerobic activity, or an equivalent combination. ^b^ FPG 100–125 mg/dL or HbA1c 5.7–6.4%, without meeting the criteria for diabetes. ^c^ FPG ≥ 126 mg/dL or HbA1c ≥ 6.5%.

**Table 4:** Participant characteristics by glycemic status: women.

	Normal	Undiagnosed Prediabetes ^b^	Undiagnosed Diabetes ^c^	*p*-Value
Number, n (weighted %)	2694 (65.4)	1562 (31.7)	161 (2.9)	
Age (years)	37.19 ± 0.29	47.53 ± 0.43	52.68 ± 1.52	0.001
BMI (kg/m^2^)	22.10 ± 0.07	23.65 ± 0.10	26.55 ± 0.63	0.001
Tooth brushing ≥ 3 times/day (%)	1632 (61.9)	849 (56.8)	63 (42.4)	0.001
Family income percentile (%)				0.001
<25	246 (9.0)	231 (12.1)	45 (23.1)	
25–50	642 (23.7)	395 (25.4)	44 (30.8)	
50–75	850 (31.8)	444 (29.8)	40 (29.0)	
≥75	922 (35.5)	483 (32.7)	29 (17.0)	
Education ≥ high school (%)	2175 (84.1)	970 (66.6)	63 (45.6)	0.001
Urban residence (%)	2300 (87.0)	1241 (82.6)	120 (75.4)	0.001
Smoking				0.227
Never	2321 (87.6)	1321 (88.8)	123 (92.3)	
Past	159 (6.4)	75 (5.4)	2 (1.1)	
Current	141 (6.1)	74 (5.8)	9 (6.6)	
Alcohol drinking (Yes, %)	1299 (51.2)	583 (43.2)	48 (34.4)	0.001
Regular exercise (Yes, %) ^a^	955 (56.4)	333 (48.2)	34 (47.4)	0.003
AST (IU/L)	18.17 ± 0.14	20.37 ± 0.27	25.56 ± 1.74	0.001
ALT (IU/L)	14.28 ± 0.18	18.28 ± 0.39	29.94 ± 3.40	0.001
FPG (mg/dL)	88.19 ± 0.14	97.75 ± 0.28	144.87 ± 4.97	0.001
HbA1c (%)	5.31 ± 0.01	5.79 ± 0.01	7.26 ± 0.13	0.001
Periodontal status				0.001
CPI 0–2	2152 (82.7)	974 (65.0)	80 (54.3)	
CPI 3	281 (9.9)	257 (17.1)	29 (15.4)	
CPI 4	261 (7.3)	331 (17.9)	52 (30.3)	

Values are weighted mean ± standard error (continuous variables) or unweighted n (weighted %) (categorical variables). *p*-Values were obtained from design-based general linear models (continuous variables) or design-adjusted chi-square tests (categorical variables). ALT, alanine aminotransferase; AST, aspartate aminotransferase; BMI, body mass index; CPI, Community Periodontal Index; FPG, fasting plasma glucose; HbA1c, glycated hemoglobin. ^a^ At least 150 min/week of moderate-intensity or 75 min/week of vigorous-intensity aerobic activity, or an equivalent combination. ^b^ FPG 100–125 mg/dL or HbA1c 5.7–6.4%, without meeting the criteria for diabetes. ^c^ FPG ≥ 126 mg/dL or HbA1c ≥ 6.5%.

**Table 5:** Association between periodontal status and undiagnosed prediabetes and diabetes.

	Undiagnosed Prediabetes			Undiagnosed Diabetes		
	Cases, n (%)	OR (95% CI)	*p*-Value	Cases, n (%)	OR (95% CI)	*p*-Value
**Men (n = 1817)**						
CPI 0–2 (n = 1085)	377 (32.6)	Reference	–	18 (1.3)	Reference	–
CPI 3 (n = 324)	159 (45.9)	1.128 (0.817–1.558)	0.462	24 (6.9)	3.594 (1.553–8.316)	0.003
CPI 4 (n = 408)	205 (48.5)	0.848 (0.593–1.213)	0.367	51 (12.2)	5.837 (2.341–14.555)	<0.001
*p* for trend			0.986			<0.001
**Women (n = 2536)**						
CPI 0–2 (n = 1835)	446 (21.4)	Reference	–	37 (1.9)	Reference	–
CPI 3 (n = 340)	133 (36.7)	1.247 (0.935–1.663)	0.133	13 (2.9)	0.843 (0.368–1.931)	0.687
CPI 4 (n = 361)	158 (41.5)	0.946 (0.633–1.415)	0.788	19 (5.3)	1.023 (0.478–2.188)	0.954
*p* for trend			0.811			0.936
*p* for interaction (sex × periodontal status)			0.916			0.004

Cases are presented as unweighted numbers (weighted prevalence, %) among participants included in the regression models (2014–2015, complete covariate data) within each periodontal status category; n indicates the number of participants in each category included in the models. Case numbers for the full study population are shown in Table 1 and Table 2. ORs are from survey-weighted multinomial logistic regression with full adjustment (Model 3; covariates listed in Section 2.4). *p* for trend was obtained by entering periodontal status as an ordinal variable. *p* for interaction was obtained by adding a sex × periodontal status interaction term to the fully adjusted model in the total study population. Regression models included participants with complete covariate data from 2014 to 2015, because regular exercise was not surveyed in 2013 (men, n = 1817; women, n = 2536). CI, confidence interval; CPI, Community Periodontal Index; OR, odds ratio.

## Data Availability

KNHANES data are publicly available from the Korea Disease Control and Prevention Agency at https://knhanes.kdca.go.kr (accessed on 4 November 2023).

## Supplementary Materials

The following supporting information can be downloaded at: https://www.mdpi.com/article/10.3390/jcm15197747/s1, Table S1: Unadjusted, age-adjusted, and fully adjusted associations between periodontal status and undiagnosed prediabetes and diabetes; Table S2: Sensitivity analyses using alternative definitions of glycemic status; STROBE checklist.
