# Supplementary material for: Association Between Periodontal Status and Undiagnosed Diabetes in Adults Without Diagnosed Chronic Diseases: The Korea National Health and Nutrition Examination Survey 2013–2015
Source: J Clin Med. 2026 Oct 8;15(19):7747. doi: 10.3390/jcm15197747 (PMC13666429; doi:10.3390/jcm15197747)
Supplement: Supplementary file 1 [file jcm-15-07747-s001.zip › jcm-4563935-supplementary.pdf]

Supplementary Materials

Association between Periodontal Status and Undiagnosed Diabetes in Adults without Diagnosed Chronic Diseases: The Korea National Health and Nutrition Examination Survey 2013–2015

Table S1. Unadjusted, age-adjusted, and fully adjusted associations between periodontal status and undiagnosed prediabetes and diabetes.

	Undiagnosed Prediabetes, OR (95% CI)			Undiagnosed Diabetes, OR (95% CI)		
	Model 1	Model 2	Model 3	Model 1	Model 2	Model 3
**Men**						
CPI 0–2	Reference	Reference	Reference	Reference	Reference	Reference
CPI 3	1.973 (1.476–2.637)	1.292 (0.931–1.793)	1.128 (0.817–1.558)	7.378 (3.431–15.864)	4.713 (2.007–11.066)	3.594 (1.553–8.316)
CPI 4	2.496 (1.881–3.311)	1.043 (0.744–1.462)	0.848 (0.593–1.213)	15.579 (7.800–31.119)	6.169 (2.408–15.805)	5.837 (2.341–14.555)
P for trend	<0.001	0.489	0.986	<0.001	<0.001	<0.001
**Women**						
CPI 0–2	Reference	Reference	Reference	Reference	Reference	Reference
CPI 3	2.172 (1.641–2.875)	1.330 (0.988–1.790)	1.247 (0.935–1.663)	1.999 (0.983–4.062)	1.168 (0.545–2.503)	0.843 (0.368–1.931)
CPI 4	2.793 (2.068–3.773)	0.959 (0.649–1.418)	0.946 (0.633–1.415)	4.062 (2.218–7.437)	1.246 (0.564–2.752)	1.023 (0.478–2.188)
P for trend	<0.001	0.713	0.811	<0.001	0.517	0.936

Survey-weighted multinomial logistic regression with normal glycemia as the reference outcome. Model 1: unadjusted. Model 2: adjusted for age. Model 3: further adjusted for body mass index, smoking status, alcohol drinking, household income, education, residential area, regular exercise, and tooth-brushing frequency (identical to Table 5). The same analytic sample with complete covariate data was used for all models (men, n = 1,817; women, n = 2,536). P for trend was obtained by entering periodontal status as an ordinal variable. CI, confidence interval; CPI, Community Periodontal Index; OR, odds ratio.

Table S2. Sensitivity analyses using alternative definitions of glycemic status (fully adjusted model).

	FPG criteria only		HbA1c criteria only		WHO IFG criterion	
	Prediabetes	Diabetes	Prediabetes	Diabetes	Prediabetes	Diabetes
**Men**						
CPI 0–2	Reference	Reference	Reference	Reference	Reference	Reference
CPI 3	1.347 (0.955–1.900)	4.730 (1.622–13.799)	1.439 (1.027–2.016)	4.257 (1.775–10.211)	1.371 (0.984–1.910)	4.019 (1.734–9.314)
CPI 4	0.978 (0.671–1.425)	7.014 (2.107–23.345)	1.150 (0.795–1.663)	6.662 (2.592–17.127)	1.164 (0.817–1.657)	5.985 (2.412–14.854)
P for trend	0.738	<0.001	0.239	<0.001	0.219	<0.001
**Women**						
CPI 0–2	Reference	Reference	Reference	Reference	Reference	Reference
CPI 3	1.492 (1.039–2.143)	1.128 (0.408–3.120)	0.953 (0.709–1.281)	0.780 (0.312–1.951)	1.015 (0.750–1.374)	0.829 (0.361–1.906)

CPI 4	1.052 (0.698–1.587)	1.164 (0.412–3.294)	0.900 (0.595–1.361)	0.977 (0.433–2.201)	0.909 (0.601–1.376)	1.009 (0.475–2.143)
P for trend	0.402	0.735	0.586	0.886	0.694	0.955

Values are ORs (95% CIs) from survey-weighted multinomial logistic regression adjusted for the same covariates as Model 3. FPG criteria only: prediabetes, FPG 100–125 mg/dL; diabetes, FPG ≥126 mg/dL. HbA1c criteria only: prediabetes, HbA1c 5.7–6.4%; diabetes, HbA1c ≥6.5%. WHO IFG criterion: prediabetes, FPG 110–125 mg/dL or HbA1c 5.7–6.4%; diabetes, FPG ≥126 mg/dL or HbA1c ≥6.5%. CI, confidence interval; CPI, Community Periodontal Index; FPG, fasting plasma glucose; HbA1c, glycated hemoglobin; IFG, impaired fasting glucose; OR, odds ratio; WHO, World Health Organization.
